# Presubiculum principal cells are preserved from degeneration in knock-in APP/TAU mouse models of Alzheimer’s disease

**DOI:** 10.1016/j.semcdb.2022.03.001

**Published:** 2023-04

**Authors:** Anam Islam, Takashi Saito, Takaomi Saido, Afia B. Ali

**Affiliations:** a UCL School of Pharmacy, 29-39 Brunswick Square, London WC1N 1AX, UK; b Department of Neurocognitive Science, Institute of Brain Science, Nagoya City University Graduate School of Medical Sciences, 1 Kawasumi, Mizuho-cho, Mizuho-ku, Nagoya, Aichi 467-8601, Japan; c RIKEN Center for Brain Science, 2-1 Hirosawa, Wako-shi, Saitama 351-0198, Japan

**Keywords:** Alzheimer’s disease, Hippocampus, Excitation, Presubiculum, Neurons, Electrophysiology

## Abstract

The presubiculum (PRS) is an integral component of the perforant pathway that has recently been recognised as a relatively unscathed region in clinical Alzheimer’s disease (AD), despite neighbouring components of the perforant pathway, CA1 and the entorhinal cortex, responsible for formation of episodic memory and storage, showing severe hallmarks of AD including, amyloid-beta (Aβ) plaques, tau tangles and marked gliosis. However, the question remains whether this anatomical resilience translates into functional resilience of the PRS neurons. Using neuroanatomy combined with whole-cell electrophysiological recordings, we investigated whether the unique spatial profile of the PRS was replicable in two knock-in mouse models of AD, *APP*^*NL-F/NL-F*^*, and APP*^*NL-F*^*/MAPT*^*HTAU*^ and whether the intrinsic properties and morphological integrity of the PRS principal neurons was maintained compared to the lateral entorhinal cortex (LEC) and hippocampal CA1 principal cells. Our data revealed an age-dependent Aβ and tau pathology with neuroinflammation in the LEC and CA1, but a presence of fleece-like Aβ deposits with an absence of tau tangles and cellular markers of gliosis in the PRS of the mouse models at 11–16 and 18–22 months. These observations were consistent in human post-mortem AD tissue. This spatial profile also correlated with functional resilience of strong burst firing PRS pyramidal cells that showed unaltered sub- and suprathreshold intrinsic biophysical membrane properties and gross morphology in the AD models that were similar to the properties of pyramidal cells recorded in age-matched wild-type mice (11–14 months). This was in contrast to the LEC and CA1 principal cells which showed altered subthreshold intrinsic properties such as a higher input resistance, longer membrane time constants and hyperexcitability in response to suprathreshold stimulation that correlated with atrophied dendrites in both AD models. In conclusion, our data show for the first time that the unique anatomical profile of the PRS constitutes a diffuse AD pathology that is correlated with the preservation of principal pyramidal cell intrinsic biophysical and morphological properties despite alteration of LEC and CA1 pyramidal cells in two distinct genetic models of AD. Understanding the underlying mechanisms of this resilience could be beneficial in preventing the spread of disease pathology before cognitive deficits are precipitated in AD.

## 1 Introduction

### 1.1 The presubiculum (PRS) is preserved anatomically in AD

Alzheimer’s disease (AD) is the most common form of dementia, majorly affecting the elderly, that is associated with neurodegeneration and progressive cognitive deficits. These deficits in AD are correlated with functional and structural alterations and are impacted from the presence of severe neuropathological lesions in the cerebrum including amyloid-β (Aβ) plaques and neurofibrillary tau tangles. This is accompanied by chronic neuroinflammation, aberrant network activity and loss of synaptic connections.

Moreover, memory loss is an early clinical feature of Alzheimer’s disease [45], and since the hippocampal region is central to learning and memory, AD pathology is more prominent in the hippocampal region [1], which consists of the hippocampal formation (dentate gyrus, the Cornu Ammonis (CA) 1–4, and the subiculum), and the parahippocampal region (PRS, parasubiculum, perirhinal, postrhinal and entorhinal cortices) [71]. These regions constitute the perforant pathway in the brain. Cells of origin of this pathway are extensively involved in neurofibrillary tangle formation and the termination zone of this pathway is known to contain numerous plaques with dystrophic neurites. Recently, the PRS has been a topic of much speculation due to its unique anatomical profile in AD. The anatomical position of the PRS imparts it a significant role in the memory formation process as it lies between the hippocampus and the entorhinal cortex thus, partaking in the input and output circuit of the hippocampal formation [16]. PRS volume loss is reported to be linked to behavioural phenotypes in AD and was suggested to be a critical region for detecting preclinical AD, connecting incipient pathology with memory function [31]. This volume loss, however, is not justified by the presence of neuropathological hallmarks, such as neuronal loss and lesions in the PRS, as it is devoid of such alterations in early-onset, late-onset sporadic and familial AD [20], [21], [22], [67]. Although it shows Aβ pathology, the amyloid there is diffuse and non-fibrillar, unlike the dense core plaques found in other hippocampal regions [46], [70]. However, whether this anatomical resilience translates into functional resilience of the PRS principal neurons is yet to be investigated.

### 1.2 Is there a spatial preservation of principal cell activity in AD?

It is also unclear which part of pathology plays a primary role in AD aetiology and continuum, although evidence strongly suggests network dysfunction plays an early neurotoxic role, triggering other pathological hallmarks thereafter [43], [49]. Severe network dysfunction is seen across the complete translational spectrum of AD from in vitro studies to animal models [43], [49], [51], [58], [7] to pre and post-symptomatic AD patients (Sperling et al. [84]; Vossel et al. [86]). The network abnormalities seen in AD constitute seizures, a high volume of hyperactive neurons showing epileptiform activity, activation and deactivation deficits, network hypersynchrony and impaired oscillatory rhythmic activity [48] and these abnormalities are especially prominent in hippocampal regions. Further, pyramidal neuron loss is a fundamental process in AD pathology that works in concert with synapse loss, altered intrinsic excitability and network alterations [14], [20], [21], [22], [67], [68], and this neuronal loss correlates well with clinical dementia [10], [13], [20], [3], [54].

Besides showing severe atrophy, excitatory cells also show hyperexcitability in terms of their biophysical and synaptic activity even during the early stages of AD [51], and this activity resembles epileptiform activity [38]. The abnormal synaptic hyperexcitability is a consistent observation that spans from human studies to animal and iPSC cell models of AD, [10], [13], [20], [23], [3], [33], [42], [54]. Also, cells exposed to amyloid pathology show reduced dendritic spine density and a surge in intrinsic cellular excitability [63], suggesting the role of amyloid in inducing hyperexcitability directly or through indirect mechanisms. The presence of tau tangles is another well-known factor that is thought to contribute to the network alterations during AD, and interestingly, spontaneous epileptiform activity seen in many familial AD mouse models has been shown to be preceded by the presence of soluble endogenous tau, thus implicating tau as a crucial piece in the network impairment puzzle [39], [4], [44]. Investigating whether the unique anatomical profile of the PRS is consistent with a preservation of the function of neurons in terms of their intrinsic biophysical properties together with their morphological preservation addresses this knowledge gap in the AD research field. Therefore, the current study aims to combine neuroanatomy and electrophysiology to investigate whether the anatomical and cellular changes associated with AD, occur in the PRS of two unique knock-in mouse models of AD, *APP*^*NL-F/NL-F*^ *and APP*^*NL-F*^ */MAPT*^*HTAU*^ and whether these changes translate into functional “preservation” of the principal cells compared with the LEC and the CA1 region of the hippocampus - the first brain regions to be significantly affected in early human idiopathic Alzheimer’s disease (AD), and in the most common familial forms of the disease [35], [52]. Exploring the functional preservation of the PRS would provide us with a better understanding of neuroprotective mechanisms in the brain that could be crucial for future therapeutic interventions in AD.

## 2 Materials and methods

### 2.1 Mouse animal procedures

All the procedures in this study were carried out in accordance with the British Home Office regulations under the Animal Scientific Procedure Act 1986, under the project licence PPL: P1ADA633A held by the principal investigator, Dr Afia Ali. All procedures were approved by both internal and external UCL ethics committees, and in accordance with the ARRIVE 2.0 guidelines for reporting experiments involving animals [18]. A total of ~100 male mice (disease models and wild-type) were used in this study. The animals were chosen randomly, and the experiments were analysed single-blinded. The animals had ad-libitum access to food and water and were reared in cages of maximum 5 inhabitants, with a day: night cycle of 12 h: 12 h.

Recently, these knock-in mouse models have been developed by Saito et al. Saito et al. [56], that produce both Aβ 42 and Aβ 40 and with a higher ratio of Aβ 42/Aβ 40, and do not rely on non-physiological promoters and present characteristic phenotypical symptoms of AD including amyloid beta pathology, neuroinflammation and memory impairment in an age-dependent manner.

The details of the two AD models include, i) *APP*^*NL-F/NF_*F^, harbouring the double Swedish KM670/671NL and Iberian I716F *APP* mutations, and ii) *APP*^*NL-F*^*/MAPT*^*HTAU*^ mouse model, harbouring human gene encoding the microtubule-associated protein *tau* (*Mapt Tau)* knock-in mice [57]. There are currently no mouse models that successfully recapitulate Aβ pathology and tauopathy as in the human condition, therefore these two models have been crossbred to study the connection between Aβ pathology and tauopathy. Although familial AD models represent approximately 5% of the human population with AD, they share similarities to the idiopathic cases, and are commonly used to make predictions regarding idiopathic cases.

The humanized MAPT in the *APP*^*NL-F*^ */MAPT*^*HTAU*^ model expressed all 6 isoforms of tau present in humans and displayed higher tau phosphorylation, cell to cell propagation of tau and presence of dystrophic neurites in the presence of Aβ plaques in all cortical areas with neuroinflammation. Propagation of tau was found not to be dependent on Aβ amyloidosis [57]. The double knock-in mice show higher tau phosphorylation along with Aβ pathology and neuroinflammation although they lack apparent tau pathology [57]. The *APP^NL-F/NL-F^* mice were genotyped via standard polymerase chain reaction using the following four primers: 5′-ATCTCGGAAGTGAAGATG-3′, 5′-TGTAGATGAGAACTTAAC-3′, 5′-ATCTCGGAAGTGAATCTA-3′, and 5′-CGTATAATGTATGCTATACGAAG-3′ as previously described [56]. While the *APP*^*NL-F*^*/MAPT*^*HTAU*^ animals were genotyped using a concoction of primers that included the above as well as Fwt5: 5′-GTCAGATCACTAGACTCAGC-3′, Rwt5: 5′-CTGTGCTCCACTGTGACTGG-3′and Rhm5: 5′-CTGCTTGAGTTATCTTGGCC-3′ [25].

### 2.2 Tissue harvesting

#### 2.2.1 Mouse brain tissue

Tissue preparation was carried out as previously described [51], [61]. All experiments were performed single-blinded. Mice were deeply anaesthetized using inhalation of isoflurane 4% followed by intraperitoneal injection of 60 mg/kg phenobarbitone. The level of anaesthesia was monitored using pedal and tail pinch reflexes, rate, depth, and pattern of respiration through observation and the colour of mucous membranes and skin. Following anaesthetisation, mice were perfused trans-cardiac with a sucrose-containing artificial cerebrospinal fluid (ACSF) solution that consisted of (in mM): 248 sucrose, 3.3 KCl, 1.4 NaH_2_PO_4_, 2.5 CaCl_2_, 1.2 MgCl_2_, 15 glucose and 25.5 NaHCO_3_, bubbled with 95% O_2_ and 5% CO_2_. The ACSF comprised of (in mM) 248 sucrose, 3.3 KCl, 1.4 NaH_2_PO_4_, 2.5 CaCl_2_, 1.2 MgCl_2_, 25.5 NaHCO_3_, and 15 glucose bubbled with 95% O_2_ and 5% CO_2_. The animals were then decapitated, the brain removed and 300 µm thick coronal sections of the cortex and hippocampus were cut in ice-cold standard ACSF using an automated vibratome (Leica, Germany). This standard ACSF contained (in mM): 121 NaCl, 2.5 KCl, 1.3 NaH2PO_4_, 2 CaCl_2_, 1 MgCl_2_, 20 glucose and 26 NaHCO_3_, equilibrated with 95% O_2_ and 5% CO_2_ (pH, 7.2–7.4, osmolarity, 300–310 mOsm).

Slices were then incubated in ACSF for 30 mins at 37 °C and transferred to room temperature prior to recording. Brain slices were placed in a submerged chamber and superfused with ACSF at a rate of 1–2 ml/min for electrophysiological recordings.

For neuroanatomical studies, one half of the brains were immediately fixed after cardiac perfusion in 4% paraformaldehyde and 0.1% glutaraldehyde in 0.1 M phosphate buffer for 24 h prior to sectioning. Coronal sectioning was performed and the entire hippocampus was sectioned at 70 µm. Posterior hippocampal sections were used for data collection from the LEC and PRS regions while anterior hippocampal sections were used for CA1 data collection from the same subject, as shown in Fig. 5 panel A. Similar sections for each region were selected for all experimental procedures including neuroanatomy and electrophysiology to maintain uniformity. Animals were excluded if a suitable region could not be retrieved due to poor histological quality. The number of animals used for each experimental procedure has been included in Tables 3 and 4.

### 2.3 Human brain tissue

A total of 14 hippocampal post-mortem brain tissue sections from 7 AD patients and 7 age-matched control individuals were obtained from Queen Square Brain Bank for Neurological Disorders, UCL Institute of Neurology according to the Human Tissue Act (HTA) 2004 and under the HTA license. Ethical approval was obtained from the local research ethics committee for the national hospital for Neurology and Neurosurgery. (Table 1).

**Table 1:** List of the human case studies used for tissue samples along with relevant details.

Cases ID	Group	Regions Used	Age (years)	Sex	Post-mortem Delay (hours)	Brain Weight (g)	Braak Staging	CERAD Score	Thal Staging
1	AD	Hippocampal region	67	Male	35.27	1223	Braak 6	CERAD 3	Thal 5
2	AD	Hippocampal region	55	Female	47.50	1100	Braak 6	Frequent	–
3	AD	Hippocampal region	90	Male	89	1200	Braak 4	CERAD 0	Thal 1
4	AD	Hippocampal region	86	Male	96.1	1203	Braak 6	CERAD 3	Thal 5
5	AD	Hippocampal region	68	Male	70.05	1522	Braak 6	–	Thal 5
6	AD	Hippocampal region	69	Male	35.04	891	Braak 6	Frequent	Thal 5
7	AD	Hippocampal region	88	Male	58.1	1084	Braak 6	–	Thal 5
8	Control	Hippocampal region	101	Male	60.35	1450	Braak 1	CERAD 0	–
9	Control	Hippocampal region	79	Male	105.5	1355	Braak 2	–	–
10	Control	Hippocampal region	88	Male	96	1240	Braak 2	CERAD 1	Thal 3
11	Control	Hippocampal region	71	Female	76	1214	Braak 3	CERAD 2	Thal 2
12	Control	Hippocampal region	86	Female	120	1234	Braak 2	–	–
13	Control	Hippocampal region	80	Female	49.10	1242	Braak 2	–	–
14	Control	Hippocampal region	83	Male	105.00	1244	Braak 4	CERAD 2	Thal 3

### 2.4 Electrophysiology

Whole-cell somatic recordings were performed using patch electrodes with resistances of 10–12 MΩ made from filamented borosilicate glass capillaries (Harvard Apparatus, UK) and filled with a solution containing (in mM): 134 K gluconate, 10 HEPES, 10 phosphocreatine, 2 Na_2_ATP, 0.2 Na_2_GTP, and 0.2% w/v biocytin (pH, 7.3, osmolarity, 300–310 mOsm). Excitatory LEC, CA1 and PRS pyramidal cells were selected for recording based on soma shape using video microscopy visualised on a monitor (Panasonic, UK) using an upright microscope (Leica, Germany) under near infrared differential interference contrast (DIC). Images were enhanced using a camera control unit (Hamamatsu, Japan). The cells were further characterized by their electrophysiological properties obtained from injecting a series of 500 ms depolarizing and hyperpolarizing current pulses.

All electrophysiological whole-cell recordings were carried out under the current clamp mode of operation (NPI SEC 05LX amplifier; NPI electronics, Germany), which allowed for the recording of the intrinsic biophysical properties of the neurons and the natural synaptic voltages to be measured; cells with stable membrane potentials were selected for experiments. Recordings were low pass filtered at 2 KHz, digitised at 5 KHz using a CED 1401 interface (Cambridge Electronic Design, UK) and fed directly to a PC running the software Signal (versions 2 and 4 used, Cambridge Electronic Design, UK). Signal was used to acquire recordings and generate currents steps.

Input resistance was monitored throughout experiments by means of a hyperpolarizing current step (−10 pA, 10 ms). Signal (Cambridge Electronic Design, UK) was used to acquire recordings and generate current steps.

**Fig. 1:**
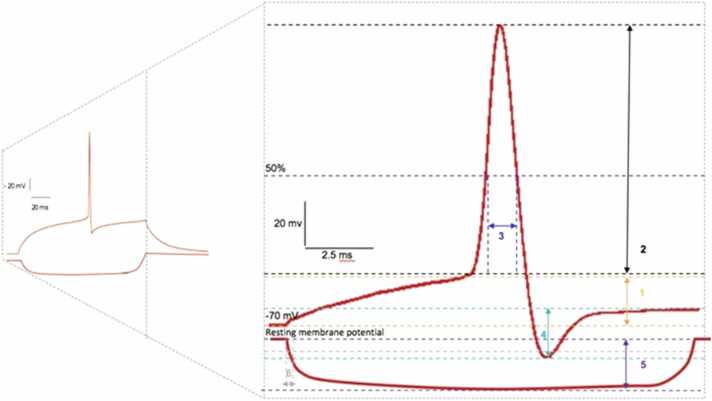
Schematic representation of the different measured parameters of an action potential. An action potential (AP) displaying: 1) AP threshold (mV) which is defined by the membrane potential (~55 mV) that a cell must reach to elicit an AP; 2) AP amplitude (mV) which measures the maximum height of an AP response; 3) half-width (ms) which measures the average time taken by an AP to reach its half amplitude; and, 4) afterhyperpolarization which is defined by the fall of the membrane potential below the resting membrane potential (~80 mV); 5) Δ of the electrotonic response (mv) which is used to calculate the input resistance in relation to the input current (na); and 6) time constant (ms), also known as tau, which measures the time taken to charge the cell membrane to elicit an AP. To prevent bias due to cell-to-cell variation in resting membrane potentials, each parameter was measured at a membrane voltage of − 65 mV, fixed with 500 ms long current injection.

Single action potential (AP) parameters were measured from responses to depolarizing current steps (+25–150 pA, 500 ms), which induced a single (shown in Fig. 1) or a, burst or trains of action potentials. After-hyperonization (AHP) amplitudes were measured from the threshold to the peak of the AHP. Any variations in the sample size for subsets of data was due to elimination of obscure or ambiguous data points presented when measuring AP threshold or AHP. From changes in voltage following a hyperpolarising current step (500 ms, with increments of −150 to −25 pA, Δ 25), the input resistance and membrane time constant were determined from voltage changes in response to hyperpolarizing current steps (−25 pA, 500 ms) from the steady state voltage change. The adaptation ratio was calculated from a train of action potentials in response to a depolarising current step (500 ms, +125 pA), by dividing the final interspike interval by the first.

### 2.5 Neuroanatomical procedures and analysis

#### 2.5.1 Recovery of biocytin-labelled cells post- electrophysiological recordings

After whole-cell patch clamp recording, immunohistochemistry was performed on the recorded neuron to study the morphological alterations of pyramidal neurons from different parts of the cerebral cortex in the limbic lobe. During whole-cell patch clamp recording, neurons were filled with biocytin. Immediately after recording, the 300 µm thick slice was fixed overnight at 4 °C in 4% paraformaldehyde solution which contained 4% paraformaldehyde (w/v) and 0.1% glutaraldehyde (v/v) in 0.1 M phosphate buffer (PB). After fixation, the slices were embedded in 12% gelatine (w/v) in dH_2_O and stored at 4 °C for 1 hr. After the gelatine solidified, the slices were carefully cut out in blocks and kept in fixative for a further 1 hr. This was done to give gelatine a firm composition for easier slicing. Once fixed, the gelatine block containing the slice was sectioned at 70 µm using a vibratome. The sections were kept in phosphate-buffered saline (PBS). Once sectioned, gelatine was carefully removed from around the slice using a dissecting microscope and scalpel blade. This was done for all the sections. The sections were incubated in freshly prepared avidin and biotinylated horseradish peroxidase complex (Vectastain ABC kit, Vectorlabs, USA) after permeabilization with Tris buffer and 0.3% (v/v) TritonX-100 (TBST 0.3%). After a 2-hr incubation, the sections were washed with TBST 0.3% and PBS. This was followed by a 20 min incubation in 3,5-diaminobenzidine (DAB) mixed with a drop of 8% nickel chloride (NiCl_2_). The reaction was oxidised with 0.2% hydrogen peroxide (H_2_O_2_). Staining was monitored under a light microscope to achieve a standard intensity across all the section and until the recorded cell became visible. The cell was stained black due to DAB- NiCl_2_ oxidation by hydrogen peroxide catalysed by the horseradish peroxidase enzyme. After reaching desired intensity, the reaction was stopped using Tris buffer. The sections were washed in Tris buffer three times, 10 min per wash. The sections were mounted on slides (Superfrost Plus, Avantor, UK) and left to dry for 24 h. The sections were then dehydrated with increasing concentrations of ethanol i.e*.,* 50%, 70%, 95%, and 100% for 10 min each with 100% incubation done twice. The sections were then transferred to Histo-Clear (National Diagnostics, UK) for clearing the sections for 10 mins. Mounting medium was used and the sections were cover slipped using Histomount mounting medium (Thermo Fisher Scientific, US) for visualisation. After recovery, the cells were reconstructed using a drawing tube mounted on a light microscope (Leica, Germany) with x100 objective for morphometric analysis. Sholl analysis was used to quantify the morphological variability in the cells and students t-test was used for statistical analysis. The confidence interval was kept at 95% (*P* < 0.05).

### 2.6 Immunohistochemical procedures and analysis

Free floating, 70 µm thick mouse brain sections were used for immunohistochemistry. The sections were permeabilized with TBST 0.3% and endogenous peroxidase was blocked with 1% (v/v) H_2_O_2_ for 30 min. Non-specific binding was blocked by 20% normal serum for 1 hr at room temperature. The sections were incubated in primary antibody (Table 2) for 48 h for immunofluorescence and 24 hr for immunoperoxidase. The sections were then washed and incubated with secondary antibodies (Table 2) for 2 hr at room temperature. For immunofluorescence, the sections were washed, counterstained with DAPI nuclear stain (1:1000) for 6–7 mins and then washed and mounted with Vectashield Antifade Mounting Medium (Vector Laboratories, UK). For immunoperoxidase, steps were followed as mentioned previously. Sections treated for immunofluorescence were imaged using a confocal laser scanning microscope (LSM 880, Zeiss, Germany). Z-stack images were taken, at three different positions within each brain section: LEC, CA1 and PRS (Brodmann area 35), 12 images per stack (1024 *1024 pixels) with x20 and x63 oil immersion objectives. Regions of interest were located using the manual joystick through the x20 objective lens by systematically searching the slice. Sections treated for immunoperoxidase were imaged using a light microscope (Leica, Germany) at x20 and x40 objectives. Image J software was used for all data acquisition. For colocalization analysis, the Coloc 2 plugin was used in Image J with Pearson’s correlation method. Analysis was preceded by selecting the region of interest in one channel of the image for accurate results. For optical density analysis, integrated density was used as a measure of quantifying pixels of interest. The triangle method was used for thresholding all images.

**Table 2:** List of antibodies and dilutions used in this study.

Antibody	Target	Manufacturer	Dilution	Host
Beta Amyloid polyclonal	C-terminal region of APP695	Thermo Fisher Scientific	1:1000	Rabbit
Phopho-Tau Monoclonal (AT8)	Human PHF Tau (Ser202/Thr205)	Thermo Fisher Scientific	1:1000	Mouse
GFAP Monoclonal	Glial fibrillary acidic protein	Thermo Fisher Scientific	1:2000	Rat
CD68 Monoclonal	Mouse microsialin	Bio-Rad	1:3000	Rat
Texas Red Anti-Rabbit	_______________	Invitrogen	1:750	Goat
Fitc Anti-Mouse	_______________	Sigma	1:200	Goat
Biotinylated Anti-Rat	_______________	Vector Laboratories	1:500	Goat

#### 2.6.1 Statistical analysis

For neuroanatomical and electrophysiology analysis, one-way ANOVA and two-way ANOVA were performed to determine statistical significance using GraphPad Prism version 8.0 for Windows. Pearson’s correlation analysis was used to assess the colocalisation between receptors and cell types using GraphPad Prism. Fisher’s Transformation was applied to Pearson’s R value to normalise the data. Data are represented as mean ± SEM. For all statistical tests performed, a 95% confidence interval was used (*P* < 0.05) and tests were one-tailed. The *“n”* is given as the number of observations and the number of animals used, unless otherwise stated. Specific statistical methods and the significance of each analysis is described in the legend of each figure.

## 3 Results

The current study aimed to investigate whether the PRS that is thought to be protected from neurodegeneration anatomically in AD, was functionally “preserved” in two mouse models that harbor the APP and APP/tau genes that will provide further insight into the spatial preservation of specific brain regions during AD pathogenesis. Here, we will present the neuroanatomical evidence of the expression pattern of APP and tau in the LEC and CA1 regions that are known to be severely affected in AD and compare these findings to the PRS region at two different age windows 11–16 months and 18–22 months, when the hallmarks of AD are expected to be fully apparent.

We then investigated the intrinsic biophysical properties of principal cells in these spatially distinct regions and correlated the membrane properties with morphometric analysis revealed by the recovered recorded cells. For these electrophysiological experiments, an age window 11–14 months was used for all genotypes due to technical difficulties associated with recording from aged tissue in vitro*.*

### 3.1 The PRS is devoid of Aβ accumulation and hyperphosphorylated tau in two AD mouse models

**Fig. 2:**
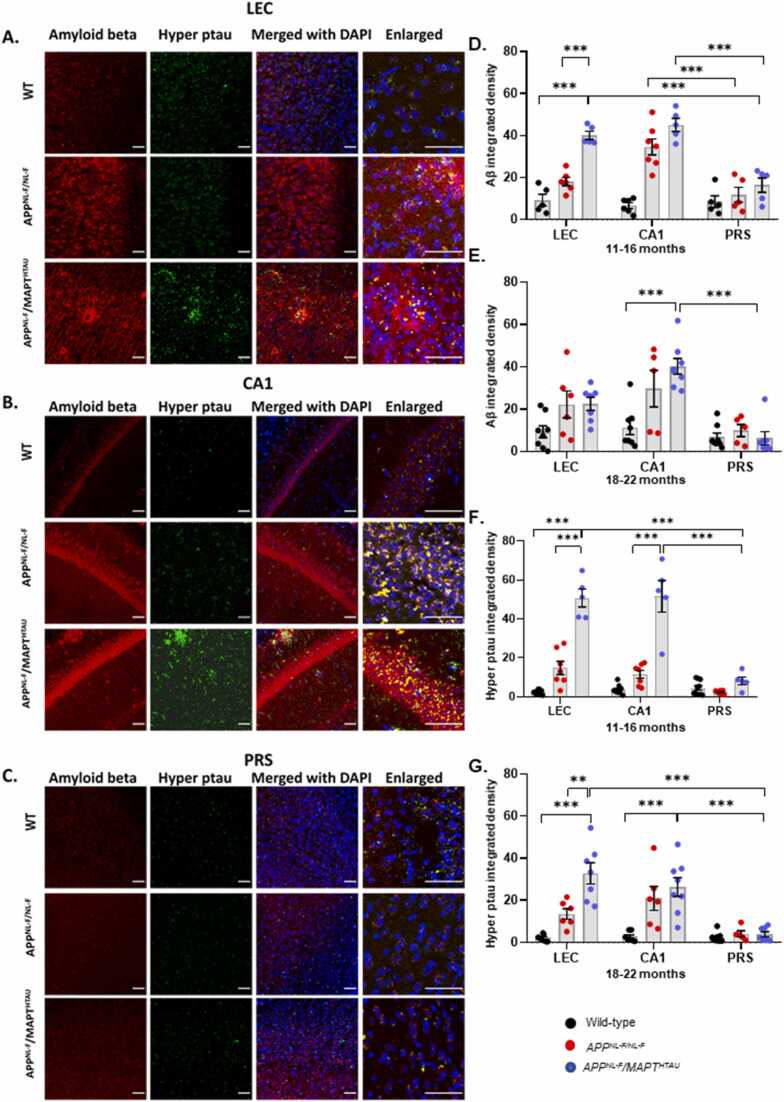
Compact amyloid-β plaques and hyperphosphorylated tau (hyper ptau) are significantly elevated in LEC and CA1, but dispersed in the PRS of knock-in APP and MAPT mouse models of AD. A- Representative confocal images taken in the LEC, CA1 and PRS respectively of 18–22-month-old wild-type, *APP*^*NL-F/NL-F*^ and *APP*^*NL-F*^*/MAPT*^*HTAU*^ mice showing amyloid-β load (red, Texas red), and hyperphosphorylated tau (green, FITC), merged with DAPI for nuclei staining (blue). Images were taken at x20 and x63 magnification (scale bar 50 µm). Arrows indicate dense-core plaques and neurofibrillary tangles D-E. Graphs showing total amyloid-β load in LEC, CA1 and PRS of 11–16-month and 18–22 month wild-type, *APP*^*NL-F/NL-F*^ and *APP*^*NL-F*^*/MAPT*^*HTAU*^ mice (Two-way ANOVA corrected for multiple comparisons with post hoc Tukey’s test. *P ≤ 0.05, **P ≤ 0.01, ***P ≤ 0.001, ****P ≤ 0.0001). F-G. Graphs showing total hyperphosphorylated tau load in LEC, CA1 and PRS of 11–16-month and 18–22-month wild type, *APP*^*NL-F/NL-F*^ and *APP*^*NL-F*^*/MAPT*^*HTAU*^ mice (Two-way ANOVA corrected for multiple comparisons with post hoc Tukey’s test. *P ≤ 0.05, **P ≤ 0.01, ***P ≤ 0.001, ****P ≤ 0.0001).

Free-floating coronal brain sections containing the hippocampal formation and the parahippocampal gyrus were immunostained for C-terminal soluble fragments of Aβ, and hyperphosphorylated tau (Fig. 2, Table 3). We found that the LEC and CA1 regions of both, 11–16-month cohort, *APP*^*NL-F/NL-F*^ and *APP*^*NL-F*^*/MAPT*^*HTAU*^ mice showed an extensive Aβ load compared to the age-matched wild type mice (Fig. 2(E)). As expected, the *APP*^*NL-F*^*/MAPT*^*HTAU*^ model showed a hyperphosphorylated tau load with significantly higher density compared to the other two genotypes (Fig. 2F)). Likewise, in the 18–22-month-old cohort, similar trends in the LEC and CA1 regions were seen, however these levels were less in comparison to the 11–16-month-old cohort (Fig. 2(G-H)). The CA1 region in particular, of both the AD models showed significantly higher Aβ density with a ~4 fold increase in *APP*^*NL-F/NL-F*^ mice and an ~5 fold increase in *APP*^*NL-F*^*/MAPT*^*HTAU*^ mice.

**Table 3:** Table gives actual values of all neuroanatomy data. All values are stated as mean ± SEM. * denotes significant difference (*P ≤ 0.05*) between wild-type control*, APP*^*NL-F/NL-F*^ and *APP*^*NL-F*^*MAPT*^*HTAU*^*values.*

Region	Age	Parameter	Wild-type		*APP*^*NL-F/NL-F*^		*APP*^*NL-F*^*/MAPT*^*HTAU*^	
			*Mean ± SEM*	*n*	*Mean ± SEM*	*n*	*Mean ± SEM*	*n*
**LEC**	(11–16 months)	Aβ integrated density	9.32 ± 2.78	5	18.11 ± 1.97	6	40.18 ± 1.93*	5
		Hyper ptau integrated density	2.39 ± 0.40	9	14.80 ± 3.40*	7	50.77 ± 4.60*	5
		GFAP optical density	4.93 ± 0.58	5	8.56 ± 2.23	5	10.23 ± 1.43	5
		CD68 optical density	1.71 ± 0.04	5	3.01 ± 0.59*	6	2.92 ± 0.59	6
	(18–22 months)	Aβ integrated density	9.36 ± 2.81	8	22.23 ± 6.35*	6	22.45 ± 3.07*	7
		Hyper ptau integrated density	2.17 ± 0.86	7	13.58 ± 2.51*	6	32.86 ± 5.03 *	7
		GFAP optical density	4.34 ± 0.60	6	7.19 ± 1.69	5	6.12 ± 0.60	6
		CD68 optical density	1.50 ± 0.29	5	2.34 ± 0.33	6	2.47 ± 0.18	7
**CA1**	(11–16 months)	Aβ integrated density	6.66 ± 1.37	5	34.64 ± 3.82*	7	45.17 ± 3.17*	5
		Hyper ptau integrated density	3.74 ± 0.78	9	11.54 ± 2.05	7	51.67 ± 8.16*	5
		GFAP optical density	10.21 ± 1.18	5	12.15 ± 3.39	6	11.91 ± 2.26	5
		CD68 optical density	1.52 ± 0.17	6	2.65 ± 0.42	6	3.53 ± 0.45*	5
	(18–22 months)	Aβ integrated density	11.39 ± 3.41	8	29.75 ± 8.64*	5	40.20 ± 3.73*	8
		Hyper ptau integrated density	2.73 ± 0.86	7	20.98 ± 5.64*	6	26.34 ± 4.44*	8
		GFAP optical density	7.72 ± 1.18	5	9.81 ± 2.53	5	12.49 ± 2.99	5
		CD68 optical density	1.84 ± 0.12	5	2.53 ± 0.51	6	3.15 ± 0.43*	7
**PRS**	(11–16 months)	Aβ integrated density	8.54 ± 2.81	5	11.87 ± 3.53	5	16.44 ± 3.46	5
		Hyper ptau integrated density	4.52 ± 1.18	10	2.30 ± 0.36	7	8.20 ± 1.97	5
		GFAP optical density	5.29 ± 1.05	5	3.37 ± 3.39	6	4.61 ± 0.83	4
		CD68 optical density	1.86 ± 0.08	6	1.85 ± 0.08	6	1.71 ± 0.07	5
	(18–22 months)	Aβ integrated density	6.79 ± 1.91	8	9.91 ± 2.86	5	6.28 ± 3.13	7
		Hyper ptau integrated density	2.47 ± 0.82	8	4.12 ± 1.44	5	3.88 ± 1.23	7
		GFAP optical density	3.83 ± 1.07	6	4.39 ± 1.54	5	2.45 ± 0.35	8
		CD68 optical density	1.86 ± 0.12	5	1.90 ± 0.26	5	1.67 ± 0.05	7

However, in stark contrast, in the PRS in both AD models, the levels of Aβ, and hyperphosphorylated tau were comparable to the age-matched wild-type mice (Fig. 2). Furthermore, the Aβ in the PRS of *APP*^*NL-F/NL-F*^ and *APP*^*NL-F*^*/MAPT*^*HTAU*^ mice had a grainy, fuzzy appearance unlike the compact morphology and frequent plaques seen in other hippocampal regions.

### 3.2 The expression of Aβ and hyperphosphorylated tau in our AD mouse models is comparable to human AD tissue

**Fig. 3:**
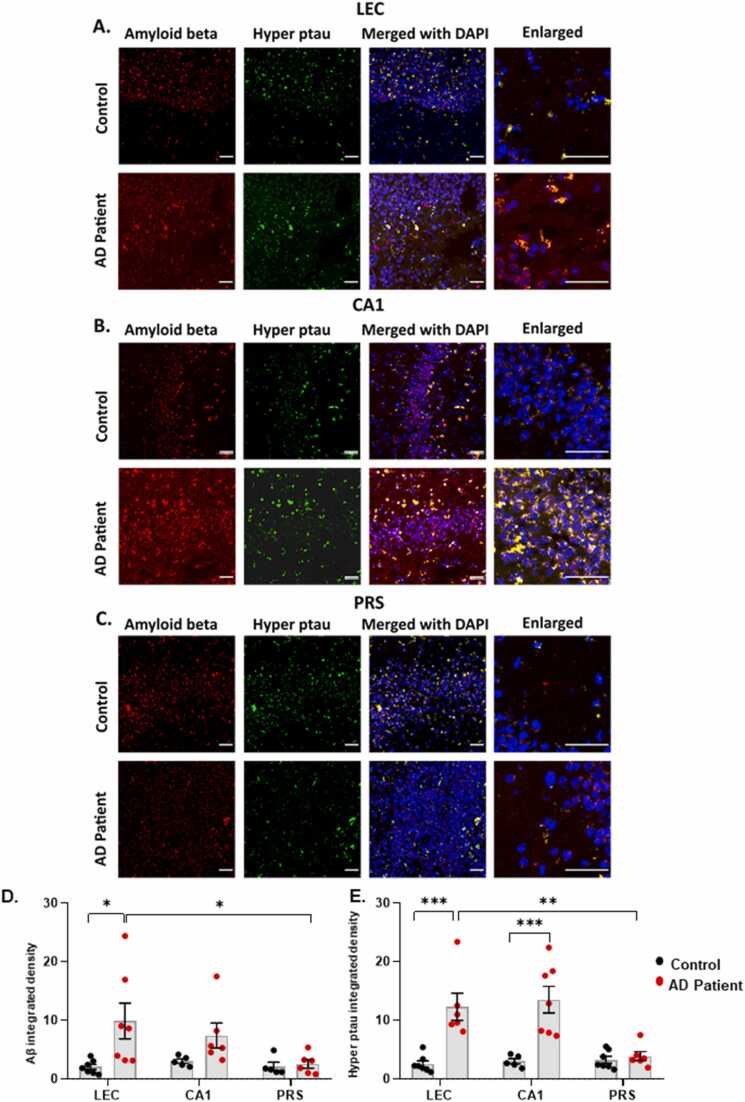
Dispersed Amyloid-β and no neurofibrillary tangles are seen in the PRS of senile AD patients. A-C. Representative confocal images taken in the LEC, CA1 and PRS respectively of post-mortem human brain tissue from control (Case ID: 11) and AD patient (Case ID: 4) showing Amyloid-β load (red, Texas red), and hyperphosphorylated tau (green, FITC), merged with DAPI for nuclei staining (blue). Images were taken at x20 and x63 magnification (scale bar 50 µm). Dense amyloid-β aggregates colocalised with tau tangles (white arrows) are seen around the CA1 and LEC of AD patients while the parvopyramidal layer of the PRS shows dispersed Aβ and an absence of tangles. D-E. Graphs showing total amyloid-β load in LEC, CA1 and PRS of control and AD patients (Two-way ANOVA corrected for multiple comparisons with post hoc Tukey’s test. * *p* < 0.033 ** *p* < 0.002 *** *p* < 0.001). F-G**.** Graphs showing total hyperphosphorylated tau load in LEC, CA1 and PRS of control and AD patients (Two-way ANOVA corrected for multiple comparisons with post-hoc Tukey’s test. * *p* < 0.033 ** *p* < 0.002 *** *p* < 0.001).

We performed comparative experiments and extended these observations in post-mortem brain tissue from confirmed cases of AD patients and found similar expression patterns for Aβ fragments and hyperphosphorylated tau in all three regions (Fig. 3, Table 3). In the LEC, there was a ~ 3-fold increase in both Aβ and hyperphosphorylated tau while the CA1 showed significant elevation of hyperphosphorylated tau when compared with control levels. In comparison to the mouse models of AD, in control and AD patients, there was no significant difference in the expression of Aβ and hyperphosphorylated tau in the PRS.

### 3.3 Neuroinflammation is virtually absent in the PRS in AD

**Fig. 4:**
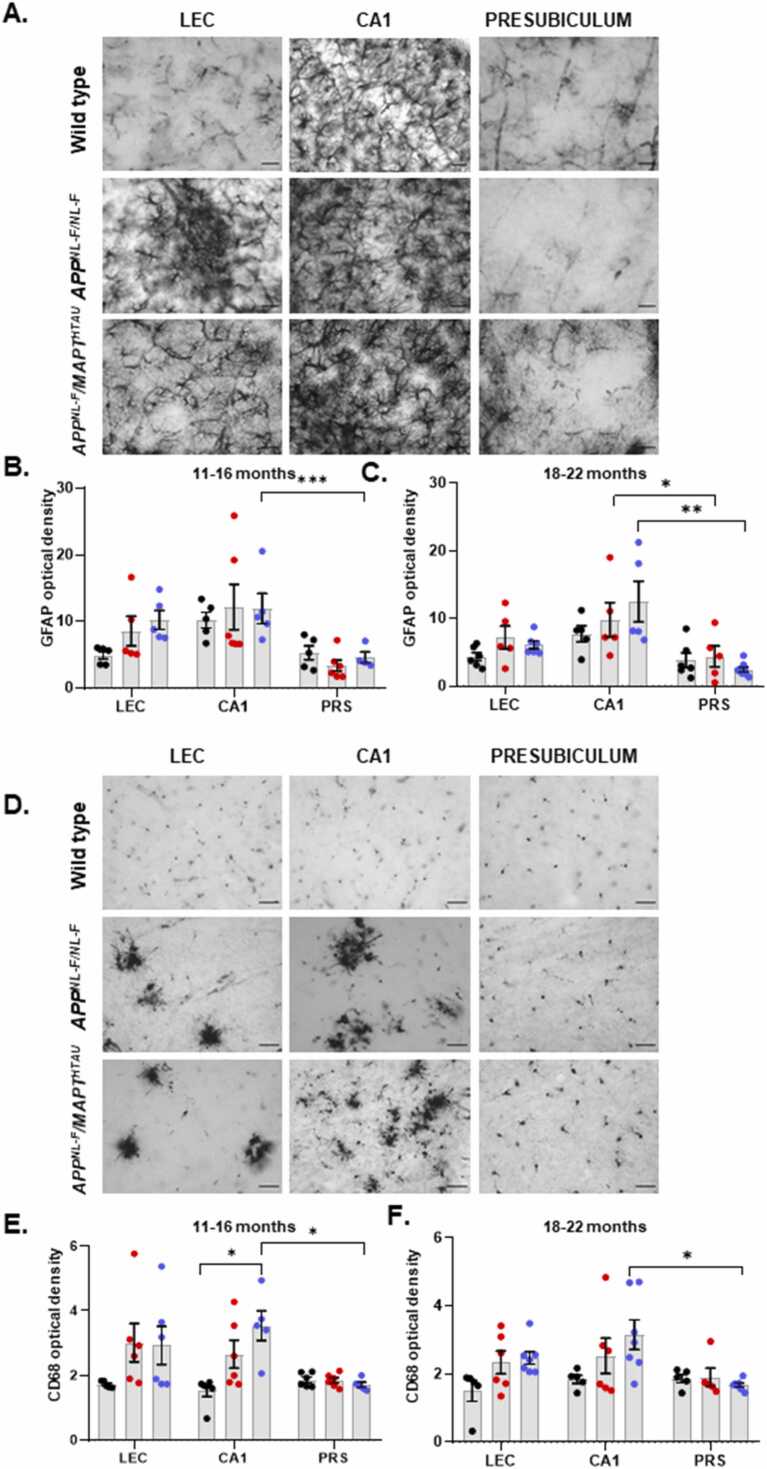
Widespread clusters of reactive astrocytes and activated microglia are seen in LEC and CA1 hippocampal regions of knock-in APP and MAPT mouse models of AD in stark contrast with the PRS. A and D) Representative brightfield images taken in the LEC, CA1 and PRS respectively of 18–22 month old wild-type, *APP*^*NL-F/NL-F*^ and *APP*^*NL-F*^*/MAPT*^*HTAU*^ mice. The LEC and CA1 of *APP*^*NL-F/NL-F*^ and *APP*^*NL-F*^*/MAPT*^*HTAU*^ mice show clusters of activated glial cells with hypertrophy, while the PRS profile is comparable to wild-type animals with virtually no compact glial formations. Images were taken at x20 and x63 magnification (scale bar 50 µm). B-C. Graphs shows quantitative data for total density of GFAP^+^ reactive astrocytes in LEC, CA1 and PRS of 11–16 month and 18–22 month old wild-type, *APP*^*NL-F/NL-F*^ and *APP*^*NL-F*^*/MAPT*^*HTAU*^ mice respectively (Two-way ANOVA corrected for multiple comparisons with post hoc Tukey’s test. *P ≤ 0.05, **P ≤ 0.01, ***P ≤ 0.001, ****P ≤ 0.0001). E-F. Graphs shows quantitative data for total density of CD68^+^ activated microglia in LEC, CA1 and PRS of 11–16 month and 18–22 month old wild-type, *APP*^*NL-F/NL-F*^ and *APP*^*NL-F*^*/MAPT*^*HTAU*^ mice respectively (Two-way ANOVA corrected for multiple comparisons with post hoc Tukey’s test. *P ≤ 0.05, **P ≤ 0.01, ***P ≤ 0.001, ****P ≤ 0.0001).

To establish whether an escalation in Aβ and hyperphosphorylated tau expression was followed by an elevation in neuroinflammatory hallmarks like activated glia, we next immunostained adjacent coronal sections for glial fibrillary acidic protein (GFAP) and cluster of differentiation 68 (CD68) (Fig. 4). Reactive astrocytes are characterised by an upregulated expression of GFAP and the GFAP marker is superior to other astrocytic markers for labelling the astrocytic processes and branches especially in the hippocampus [74]. In both, 11–16-month cohorts there was an upregulation in GFAP^+^ astrocytes and CD68^+^ microglia in both the LEC and CA1 regions in both models of AD, compared to age-matched wild type animals. In the 18–22-month-old mice, there was marked gliosis, signifying proliferation and hypertrophy, that was extensive with the presence of distinct clumps (Fig. 3), however these levels were proportionately less compared to the 11–16-month-old cohorts (see Table 3). Interestingly, the presence of activated microglia did not necessarily signify an increase in cell numbers. Rather, the activated glia were associated with an altered phenotype unlike the glia present in age-matched wild-type animals, showing hypertrophy with thickened processes in the case of astrocytes and hyper-ramified, swollen large body and thick short processes in the case of microglia. In stark contrast to LEC and CA1, the PRS region in both the disease models showed no signs of activated glia and was virtually void of GFAP^+^ and CD68^+^ cells.

### 3.4 The membrane input resistance and time constant of principal neurons increased in both AD models

Whole-cell patch clamp recordings were performed on somas of 23 wild-type, 21 *APP*^*NL-F/NL-F*^*, and 21 APP*^*NL-F*^*/MAPT*^*HTAU*^ pyramidal cells in the three regions (only identified cells includes in this study, see Table 4 for details) and included cells with stable resting membrane potentials (RMP).

**Table 4:** presents mean ± SEM of membrane biophysical properties in response to either − 25 pA hyperpolarising or + 25–50 pA depolarising current injections elicited in principal cells recorded in the LEC, CA1 and PRS in 12–14 months old wild-type, *APP*^*NL-F/NL-F*^*and APP*^*NLF*^*/MAPT*^*HTAU*^*mice.* RPM (resting membrane potential), AP (action potential), Amp (Amplitude), HW (half-width), fAHP (fast after-hyperpolarisation), R (resistance). (Two-way ANOVA with Tukey’s multiple comparison test used for RMP, time constant and input resistance analysis, for rest One-way ANOVA with Tukey’s multiple comparison test used. * denotes significant difference (*P ≤ 0.05*) between wild-type control*, APP*^*NL-F/NL-F*^ and *APP*^*NL-F*^*MAPT*^*HTAU*^*values.*

Region	Biophysical Parameter	Wild-type		*APP*^*NL-F/NL-F*^		*APP*^*NL-F*^*/MAPT*^*HTAU*^	
		*Mean ± SEM*	*n*	*Mean ± SEM*	*n*	*Mean ± SEM*	*n*
**LEC**	RMP (mV)	-65.28 ± 0.83	7	-61.40 ± 1.03*	6	-59.14 ± 0.93*	7
	AP threshold (mV)	24.42 ± 1.42	7	20.83 ± 1.13	6	18.95 ± 1.79*	7
	AP Amp (mV)	80.16 ± 0.98	7	80 ± 0.57	6	81 ± 0.92	7
	AP width (ms)	2.04 ± 0.10	7	1.85 ± 0.07	6	1.78 ± 0.05	7
	fAHP Amp (mV)	3.74 ± 0.45	7	3.86 ± 0.39	6	4.10 ± 0.18	7
	fAHP width (ms)	4.28 ± 0.71	7	4.33 ± 0.80	6	3.93 ± 0.49	7
	Input R (MΩ)	544 ± 17.16	7	625.00 ± 17.07*	6	622.85 ± 19.36*	7
	Time constant	14 ± 1.06	7	19.16 ± 0.94*	6	18.71 ± 0.60*	7
**CA1**	RMP (mV)	-64.44 ± 1.34	9	-59.87 ± 1.09*	8	-61.28 ± 1.14	7
	AP threshold (mV)	19.33 ± 1.29	9	16.28 ± 1.96	7	24.42 ± 1.42	7
	AP Amp (mV)	81 ± 1.29	9	83.87 ± 2.27	8	81.14 ± 1.05	7
	AP width (ms)	2.37 ± 0.08	9	2.12 ± 0.05	8	2.14 ± 0.15	7
	fAHP Amp	4.51 ± 0.34	9	3.91 ± 0.44	8	3.71 ± 0.81	7
	fAHP width (mV)	3.33 ± 0.14		4.04 ± 0.19	7	3.70 ± 0.33	7
	Input R (MΩ)	369 ± 25.59	9	516.25 ± 19.63*	8	500 ± 20.70*	7
	Time constant	12 ± 0.55	9	22.12 ± 0.58*	8	21.57 ± 0.61*	7
**PRS**	RMP (mV)	-63.71 ± 0.60	7	-64.28 ± 0.96	7	-63.14 ± 0.91	7
	AP threshold (mV)	16.28 ± 1.96	7	17.42 ± 1.34	7	18.14 ± 2.05	7
	AP Amp (mV)	83.28 ± 1.91	7	81 ± 1.7	7	82 ± 1.74	7
	AP width (ms)	1.65 ± 0.04	7	1.65 ± 0.07	7	1.64 ± 0.07	7
	fAHP Amp (mV)	2.92 ± 0.27	7	3.28 ± 0.21	7	3.18 ± 0.32	7
	fAHP width (ms)	3.07 ± 0.07	7	2.85 ± 0.14	7	2.42 ± 0.20	7
	Input R (MΩ)	321 ± 17.71	7	284 ± 10.13	7	308 ± 12.61	7
	Time constant	10.71 ± 0.47	7	10.00 ± 0.53	7	10.57 ± 0.52	7

**Fig. 5:**
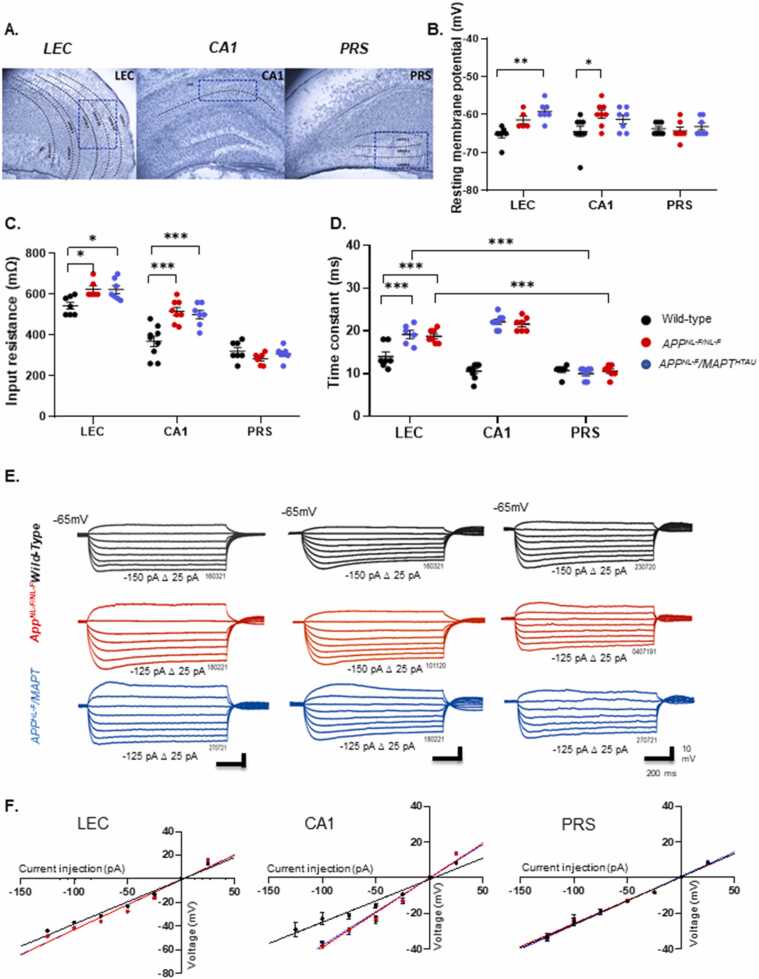
The input resistance and time constant of principal pyramidal cells increase in the LEC and CA1 but not in PRS principal cells during AD progression. A) Panel shows layer specific cytoarchitecture of LEC, CA1, PRS hippocampal regions. Cells for whole cell patch clamp recordings were selected from layer 2 and 3 of the LEC and PRS and stratum pyramidale of CA1 regions demarcated in blue. **B-D)** Graphs detail the changes in the individual cell resting membrane potential, membrane input resistance and membrane time constant for individual pyramidal cells in the three spatial regions recorded in wild-type and *APP*^*NL-F/NL-F*^ and *APP*^*NL-F*^*/MAPT*^*HTAU*^ mice at 11–16 months of age. Cell input resistance and time constant were significantly higher in both LEC and CA1 in *APP*^*NL-F/NL-F*^ and *APP*^*NL-F*^*/MAPT*^*HTAU*^ cohorts compared to wild-type mice. (*n ≥ 6 per cohort,* Two-way ANOVA corrected for multiple comparisons with post hoc Tukey’s test. *P ≤ 0.05, **P ≤ 0.01, ***P ≤ 0.001, ****P ≤ 0.0001) **E)** Example voltage responses to various current steps in LEC, CA1 and PRS pyramidal cells to 500 ms step current injections ranging from − 125–50 pA in 25 pA increments. **F)***I-V* plot of subthreshold responses in all pyramidal cells recorded in different brain regions and genotypes. The input-output curves displayed a linear relationship (*n ≥ 5* cells per cohort) (One-way ANOVA with Tukey’s multiple comparison test, *P ≤ 0.05, **P ≤ 0.01, ***P ≤ 0.001, ****P ≤ 0.0001).

The aim from the electrophysiology experiments was to investigate any alteration in the sub- and suprathreshold intrinsic membrane properties of PRS pyramidal cells compared to LEC and CA1 pyramidal cells in three genotypes. In the LEC and CA1, there were significant changes in the RMP, membrane input resistance and membrane time constants equally in both AD models, while no significant changes were seen in the PRS cells recorded and membrane potentials were close to − 63 mV (see Table 4, Fig. 5(B-D)).

All recorded cells displayed a linear voltage change to direct positive subthreshold somatic current injection (Fig. 5(E-F)). A “sag” and rebound depolarisation in response to a large hyperpolarising current step was apparent in ~50% of the total CA1 and LEC cells recorded; however, no such prominent “sag” was observed in the population of cells studied in the PRS groups. The sag and depolarizing rebound potential in response to a fixed negative current step were not significantly different between the three genotypes and was not investigated further due to the low sample range.

**Fig. 6:**
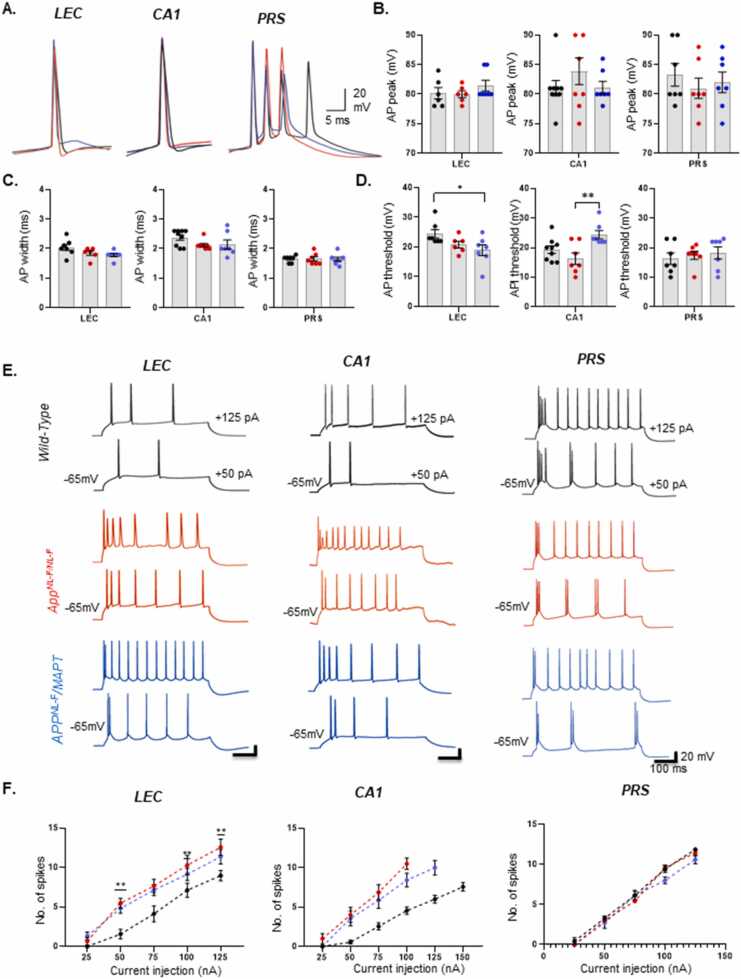
The PRS principal cells are resilient to membrane hyperexcitability during AD progression. Voltage-independence of bursting in perforated-patch recordings. A) Superimposed pyramidal cell APs recorded from wild-type, *APP*^*NL-F/NL-F*^ and *APP*^*NL-F*^*/MAPT*^*HTAU*^ mice in response to the first suprathreshold current injection of 25 pA. The LEC and CA1 cells responded with a tonic AP, while, PRS cells often displayed a strong burst with decreasing AP amplitudes. B-D) Graphical representation of the cell-to-cell AP peak amplitude, with (measured at half amplitude) and AP threshold to firing in pyramidal cells recorded in the three regions and genotypes. These properties were not significantly dependent on genotype or spatial region apart from AP width, which decreased in *APP*^*NL-F/NL-F*^ and *APP*^*NL-F*^*/MAPT*^*HTAU*^ mice in LEC and CA1 only. E) Example voltage response to suprathreshold current injection in pyramidal cells recorded in LEC, CA1 and PRS in the three genotypes. F) current / voltage relation to increasing suprathreshold current injection in all pyramidal cells recorded. In general, the input-output curves displayed a pseudo-linear relationship between number of spikes generated with increasing current injections in wild-type and the AD models. The membrane of the pyramidal cells in *APP*^*NL-F/NL-F*^ and *APP*^*NL-F*^*/MAPT*^*HTAU*^ mice in LEC and CA1 was more excitable compared to wild-type mice; furthermore, PRS cell firing was not affected by genotype. One-way ANOVA with post hoc Tukey’s test for multiple comparisons, (*n ≥ 6* cells per cohort, One-way ANOVA with Tukey’s multiple comparison test, *P ≤ 0.05, **P ≤ 0.01, ***P ≤ 0.001, ****P ≤ 0.0001).

LEC pyramidal cells in this study had, on average, the highest mean input resistance in wild-type mice which was, 544 ± 17.16 MΩ and also the slowest time constant of 14 ± 1.06 ms. Both of these parameters significantly increased by, 14.82 ± 2.20% and 36.85 ± 0.25% of control, respectively in *APP*^*NL-F/NL-F*^ *mice and* by 14.27 ± 2.411% and 33.64 ± 0.15% in *APP*^*NL-F*^*/MAPT*^*HTAU*^ mice, respectively (*P* ≤ 0.05 0.001). Similarly, the input resistance and time constant increased in CA1 pyramidal cells compared to the age-matched wild-type cells, by 43 ± 5.90% and 84.33 ± 0.26%, respectively in *APPNL*^*-F/NL-F*^ mice and by, 39.78 ± 5.89% and 79.75 ± 0.27% in *APP*^*NL-F*^*/MAPT*^*HTAU*^ mice, respectively (*P* < 0.001). There was no significant change in these parameters in the PRS cells recorded compared to the control wild-type mice (Table 4). The increase in input resistance was reflective of a larger change in membrane voltage per current step, and consistent with the increase in the time constant, favouring increased excitability for the same injected positive current step. This was also consistent with the decrease in firing threshold in both LEC and CA1 pyramidal cells of *APP*^*NL-F/NL-F*^*, and APP*^*NL-F*^*/MAPT*^*HTAU*^ mice compared to wild-type litter mates, which was a decrease by, 14.07 ± 0.19% and 22.39 ± 0.51%, respectively (Fig. 6(D)).

### 3.5 Single AP characteristics are altered in knock-in APP and Tau models

Table 4 shows the average changes in the biophysical properties recorded in the three regions and genotypes. It was difficult to get a single AP response from PRS cells, due to the initial burst, therefore measurements were taken from the first AP from the burst (Fig. 6(A-D)).

There was no significant difference in the AP amplitude, fast AHP properties between the three genotypes or region. However, there was a trend in narrowing of the AP width recorded in pyramidal cells in the AD models compared pyramidal cells recorded in wild-type mice (measured at half amplitude), but there was significant difference between the genotypes. In *APP*^*NL-F/NL-F*^ *mice,* AP narrowed by 9.31 ± 0.007% in LEC, and 10.63 ± 0.006% in CA1, (*P ≥ 0.05),* and *in APP*^*NL-F*^*/MAPT*^*HTAU*^ mice, AP narrowed by 12.45 ± 0.007% in LEC and 9.88 ± 0.02% in CA1 (*P ≥ 0.05)*. Furthermore, no significant differences were observed in the AP or fast AHP properties in the PRS pyramidal cells recorded between the three genotypes.

### 3.6 PRS principal cell firing frequency was not altered in the AD models

In response to suprathreshold somatic positive current injections, all cells displayed spike frequency adaptation and accommodation (Fig. 6(E-F)). In general, the LEC and CA1 showed a single or double AP with the same magnitude of depolarising current injection, some weak burst-firing cells (two or more APs) were observed in these regions, however, the PRS cells consistently responded with a strong burst of APs to initial depolarisation. A strong burst was defined as three or more APs elicited by short interspike intervals within 10 ms, which was followed by more than one burst and/or single APs with increasing current injection. Within a burst, successive APs declined in amplitude.

On average the firing frequency of LEC and CA1 cells overall generated a regular train of APs and that fired in a frequency range of ~ 10–15 Hz in wild-type mice. The train of APs had an adaption ratio, calculated from a train of action potentials in response to a depolarising current step (500 ms, +125 pA), by dividing the final interspike interval by the first, which was in the range of 2 and 4 in wild-type cells mice. The ratio increased similarly in both mouse models and was in the range of 6 and 10.

The PRS cells, most of the pyramidal cells responded with bursts of APs that showed characteristic firing patterns that resembled chattering cells reported previously in the superficial pyramidal cell layer of the cortex that fired repetitive synchronous bursts of rhythmic APs in the gamma frequency band of ~ 20–100 Hz [24] and similar to pyramidal cells in the subiculum [64]. The PRS cells recorded in *APP*^*NL-F/NL-F*^*, APP*^*NL-F*^*/MAPT*^*HTAU*^ and wild-type mice in our experiments, fired at a frequency of ~25–30 Hz. The mean adaptation ratios for PRS cells, (calculated from a train APs, by dividing the final interspike interval by the first after the initial burst), were in the range of 0.8–1.6, and were similar amongst the three different genotypes.

The striking differences was the membrane hyperexcitability, indicated by the greater sensitivity to intracellular positive current injection observed in pyramidal cells recorded in LEC and CA1, in both AD models which was consistent with our previous report [50]. The present study, however, did not include cells that showed a gradual deterioration in membrane resistance and time constant and the inability of the neurons to fire action potentials, which was reported in the *App*^*NL-F/NL-F*^ mice of > 18 months of age. The introduction of the tau gene did not therefore alter the firing frequency, and the membrane hyperexcitability was characteristically absent in the PRS region (Fig. 6(A & B)).

### 3.7 PRS principal excitatory cells show preserved dendritic arborization in the *APP*^*NL-F/NL-F*^ and *APP*^*NL-F*^*/MAPT*^*HTAU*^ mice

**Fig. 7:**
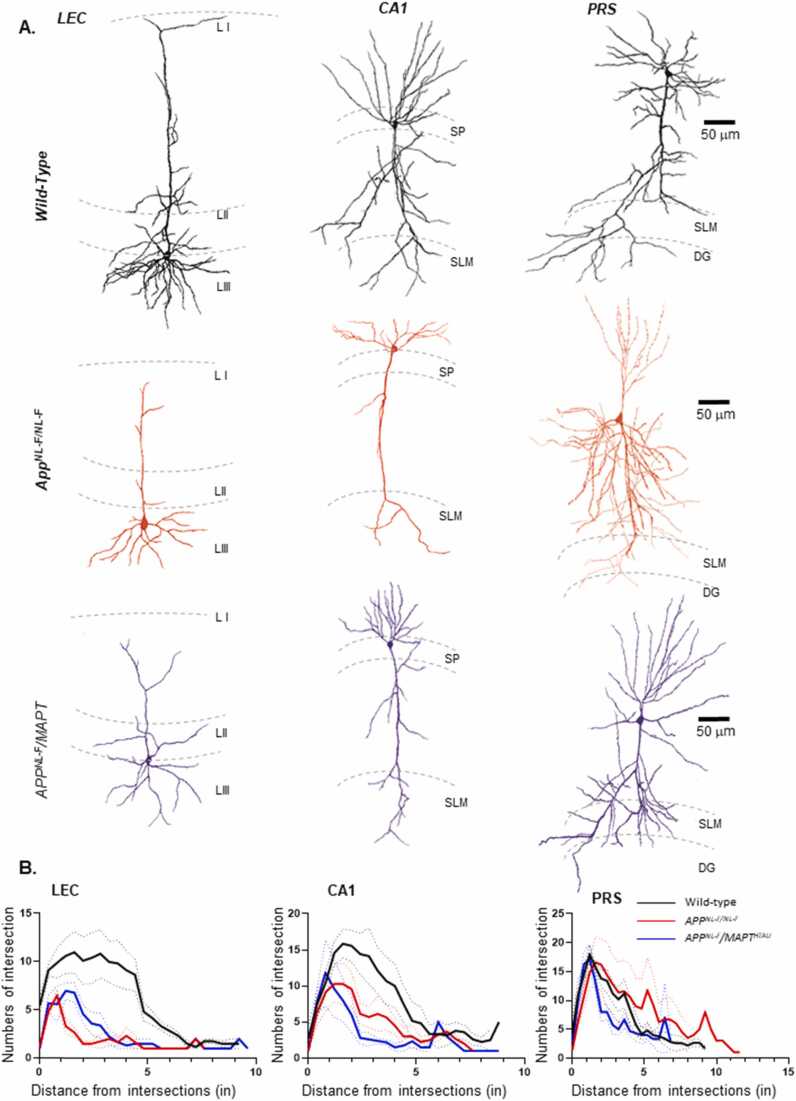
PRS principal cells show preserved morphology and dendritic complexity in contrast to LEC and CA1 pyramidal cells in knock-in APP and MAPT mouse models of AD. A. Morphological reconstruction of soma and dendrites of pyramidal cells in LEC, CA1 and PRS from wild-type, *APP*^*NL-F/NL-F*^ and *APP*^*NL-F*^*/MAPT*^*HTAU*^ mice and using a drawing tube and light microscope under x100 magnification. The PRS cells are comparable in all three genotypes. B-D. Sholl plots exhibiting the pattern of dendritic arborisation of reconstructed pyramidal cells in wild type, *APP*^*NL-F/NL-F*^ and *APP*^*NL-F*^*/MAPT*^*HTAU*^ mice in three brain regions i.e*.,* LEC, CA1 and PRS respectively (*n ≥ 5* per cohort). The sholl profile shows mean number of intersections of dendrites as a function of distance from the soma (μm). Results are expressed as mean ± SEM values. SLM (stratum lacunosum moleculare), SO (stratum oreins), SP (stratum pyramidale), DG (dentate gyrus).

During whole-cell recordings, the cells were filled with biocytin and then recovered and reconstructed using a light microscope under x1000 magnification (Fig. 7). This was to ensure that all recorded cells were indeed principal cells and that a morphometric analysis could be performed using the Sholl analysis plugin as previously described [19], with the Fiji software [59]. The PRS cells were not altered anatomically, whereas statistically significant differences were found in the gross morphology between pyramidal cells recorded in LEC and CA1 regions of both AD models compared to age-matched wild-type cells, which was consistent with the biophysical changes observed in these regions described above (Fig. 7(A-B)).

The pyramidal cells recorded in layer 3 of the LEC, shared a neuroanatomical resemblance with that of the CA1 pyramidal cells recorded in the stratum pyramidale or slightly displaced in stratum oriens. All of these cells had a distinct triangular shaped soma from the apex of which arose a single dendrite that further divided into smaller branches. They were also composed of basal dendrites which emanated from the base of the soma. The PRS cell dendrites would often terminate in the dentate gyrus.

All principal cells, their spatial location, had spiny dendrites, which is one of the most profound features of the principal pyramidal cells which increases their surface area. The axon of all principal cells (not reconstructed), was a thread-like structure, projected from the base of the soma and ramified across layers.

The sholl profile of wild-type animals in all three regions revealed a high dendritic arborisation compared with age-matched *APP*^*NL-F/NL-F*^ and *APP*^*NL-F*^*/MAPT*^*HTAU*^ animals. In general, PRS principal excitatory cell ramifications were spread out over a broader length in all three genotypes. The total mean number of intersections (within 400 µm^2^) of principal cells in *APP*^*NL-F*^*/MAPT*^*HTAU*^ mice was significantly lower than wild-type mice in both LEC and CA1 regions. Total mean intersections within the range of 400 µm^2^ in LEC were 134.13 ± 1.45 in wild-type mice and 51.41 ± 0.62 for *APP*^*NL-F*^*/MAPT*^*HTAU*^, a decrease of 61.66 ± 1.00% (*n ≥ 5, p < 0.001*) while a decrease of 54.26 ± 1.41% was seen in the CA1 (wild-type: 159.13 ± 2.24; *APP*^*NL-F*^*/MAPT*^*HTAU*^*:* 72.78 ± 1.18*; n ≥ 5; p = 0.001*). Consequently, the area under the intersection curve (AUC; in arbitrary units) of *APP*^*NL-F*^*/MAPT*^*HTAU*^ mice was also lower (wild-type: 3216 ± 310.2; *APP*^*NL-F*^*/MAPT*^*HTAU*^*:* 1367 ± 119.1; *n ≥ 5*). In the LEC, all cells were ramified closer to the soma in all three genotypes. For *APP*^*NL-F/NL-F*^ mice there was a significant reduction of 72.47 ± 0.92% in the total number of mean intersections in the LEC (*n ≥ 5, p < 0.001*). A similar trend was observed in the CA1 region of both the disease models compared to age-matched wild-type animals. Ramifications were spread out, but not as much as seen in the PRS. Consequently, a marked reduction was also visible in the spine density in LEC and CA1 principal cells of both the disease models compared to age-matched wild-type animals. Spine are crucial for excitatory inputs and the loss of which correlates directly with loss of synaptic function [17]. We did not quantified the changes in spine densities here, but others have used sophisticated in vivo two photon imaging technology to investigate this in transgenic mouse models where presence of intra and extracellular plaques correlated with reduced spine density and formation in layer V pyramidal neurons [75].

Overall, the sholl curve representing pyramidal cells from the AD models in CA1 and LEC appeared to be shifted left- and downwards, when compared to the control curve, a shift that indicated a reduction in dendritic complexity from mid- to peripheral parts of the dendritic tree.

## 4 Discussion

The PRS, a brain region that lies between the entorhinal cortex and the hippocampus, is curiously ‘anatomically preserved’ from neurodegeneration in human post-mortem brain samples from sporadic AD and familial AD; this is despite neighbouring CA1 and the lateral entorhinal cortex regions that undergo severe AD-related destruction. In this study we investigated whether these observations extended in two mouse models of AD and examined the effects of tau on Aβ load and the causal effects it may have on the alteration of neuronal anatomy and intrinsic membrane properties of principal cells in the PRS. Using two familial knock-in mouse models of AD that harbour the homozygous human APP gene or a combination of human APP and MAPT genes, we described interrelated spatially and temporally distinct anatomical and neurophysiological correlations in Aβ and tau pathology with neuronal survival in three cortical regions. These included the following: i) a differential expression pattern and propagation of Aβ and hyperphosphorylated tau pathology as seen in human AD patients. Aβ load, hyperphosphorylated tau fibrils and neuroinflammatory hallmarks appeared in the same cortical regions i.e*.,* transentorhinal and limbic regions and showed similar sequential and temporal progression as in AD, attaining Braak stage IV in the oldest animals. For this part of the study, two different age groups were chosen in order to explore the sequence of events leading to end-stage neuropathology in these models and we compare these findings to human AD brain tissue studies. ii) Functional and morphological resilience of principal cells in the PRS region in both, the *APP*^*NL-F*^*/MAPT*^*HTAU*^ and *APP*^*NL-F/NL-F*^ models during AD pathogenesis was observed. Excitatory pyramidal cells of the PRS region displayed normal intrinsic electrical membrane properties, suggesting a functional preservation, unlike the pyramidal cells recorded in the LEC and CA1 regions.

### 4.1 Spatially distinct propagation of Aβ and hyperphosphorylated tau pathology

We found a widespread increase in total Aβ and hyperphosphorylated tau load in the lateral entorhinal cortex and CA1 regions of both age groups in the *APP*^*NL-F*^*/MAPT*^*HTAU*^ mouse model compared to the age-matched wild-type and *APP*^*NL-F/NL-F*^ mice. The density of these pathological proteins was substantially more in the 11–16-month-old group. Moreover, levels of Aβ and hyperphosphorylated tau were similar to control in the PRS in both *APP*^*NL-F/NL-F*^ and *APP*^*NL-F*^*/MAPT*^*HTAU*^ mice and different aged cohorts. Hyperphosphorylated tau was virtually absent in the PRS. The older age group of 18–22 months *APP*^*NL-F*^*/MAPT*^*HTAU*^ mice showed slightly reduced Aβ and hyperphosphorylated tau soluble aggregates compared to the younger cohort in LEC and CA1, with the expression of frequent dense-core plaques and tangles with dystrophic neurites, which represent the mature end-stage of these abnormal proteins. These end-stage plaques and tau tangles were virtually absent from the 11–16 months old *APP*^*NL-F*^*/MAPT*^*HTAU*^ mice compared to the age-matched wild-type animals. Analysis of human post-mortem brain tissue from AD patients revealed a similar profile as seen in the 18–22 months old group of the *APP*^*NL-F*^*/MAPT*^*HTAU*^ mouse model.

Our results build upon previous studies which show an absence of pathological lesions in the PRS [46], [70]. A grainy fuzzy profile of Aβ was observed in this region, suggesting that PRS Aβ does not evolve into plaques with dystrophic neurites, even in the older group of both the disease models, unlike the other hippocampal regions. This is in stark contrast to the surrounding entorhinal cortex and subiculum regions which show severe tau and amyloid pathology along with neuronal atrophy [70]. This is also reported by multiple studies using immunohistochemical and PET imaging techniques ([46], [70]; Ikonomovic et al. [80]). Diffuse plaques of the presubiculum although amorphous in nature are still composed of Aβ 1–42, a component of the mature plaques in other regions. Despite this, the amorphous plaques of the presubiculum do not transform to mature senile plaques even in the late stages of the disease [46]. This lack of fibrilization is speculated to be caused by a lack of fibrillization-causing proteins such as APOE that are released by activated astrocytes [60] and severe gliosis [70]. This is further supported by the presence of diffuse amyloid plaques in the brains of cognitively normal individuals [15]. It is interesting to note that the diffuse-like cellular pathology of amyloid is not just limited to Alzheimer’s disease, as one study reported similar diffuse lake-like deposits in familial and sporadic Alzheimer disease, familial Danish dementia and familial British dementia [46]. Experiments to find a difference in the molecular pathways between the PRS and surrounding entorhinal cortex found a lack of Aβ residues terminating at residue 40 and small quantities of pyroglutamate modified Aβ [46]. Since pyroglutamate modifications are associated with an increased tendency to aggregate, it could be speculated that lack of these modifications is behind the fuzzy appearance of Aβ in the PRS. Differential processing of the Aβ peptide via the β- and γ-secretases was ruled out as a possible mechanism behind the unique PRS anatomy as the PRS region of three different neurodegenerative diseases such as AD, familial British dementia, and familial Danish dementia was shown to be similarly preserved despite the presence of different protein aggregates [46]. Thus, this unique anatomy could likely be due to the local anatomical features of the PRS than the processing of Aβ peptide. Absence of neuropsychological studies targeting the role of PRS confound the understanding of the anatomical, functional and behavioral correlation resulting from this unique phenomenon. The PRS region is associated with scene-based cognition and spatial navigation. One study found compromised scene reconstruction including spatial coherence however the authors concluded that posterior cingulate gyrus was prominently involved in both the experimental groups i.e., AD and control [28]. Another study showed navigational deficits in both the aged and AD groups compared to young and MCI patient groups ascertained through the use of virtual reality [53], however a direct correlation between the different regions and this apparent deficit was not established.

Despite the lack of anatomical alterations, PRS volume is affected early in the disease and could be used as a diagnostic marker for detecting presymptomatic AD ([31]; Carlesimo et al. [77]) as it correlated with performance on memory tests in patients with mild cognitive impairment. It could be speculated that shrinkage of the PRS volume despite lack of neuropathological lesions and neuronal loss, could be due to less projections along the perforant pathway caused by entorhinal tau and hippocampal amyloid disruption. The lack of hyperphosphorylated tau pathology in this region could be explained similarly where reduced projections to the PRS could possibly stop the seeding and propagation effects of pathological tau.

The exaggerated profile of hyperphosphorylated tau and Aβ in both the LEC and CA1 of *APP*^*NL-F*^*/MAPT*^*HTAU*^ mouse model could be attributed to the humanization of the murine tau gene. This is because pathological tau better interrelates with human tau in vivo and expresses all six isoforms after alternative splicing, unlike the three isoforms found with murine tau. Additionally, the presence of Aβ upregulates the formation of pathological tau which further triggers the accumulation of Aβ fibrils contributing to a vicious cycle. This is corroborated by previous studies which show increased seeding and trafficking of pathological tau in the presence of Aβ [2], [26]. Beyond plaques and tangles, physiological Aβ and tau have been shown to interact at endogenous levels in humans [2]. Thus, it may be speculated that this interaction becomes hypersensitive in the disease state such that it creates a sufficient microenvironment for abnormal protein seeding. This could also explain the slight increase in hyperphosphorylated tau load seen in *APP*^*NL-F/NL-F*^ mice, where murine tau is expressed at endogenous levels compared to the age-matched wild-type mice. The unique increase of soluble Aβ and tau aggregates in the 11–16-month-old age group of the *APP*^*NL-F*^*/MAPT*^*HTAU*^ mouse model compared to the old age group indicates that these pathological changes occur early in the disease aetiology, and they lessen with the disease continuum, transforming into aggregated forms that are aptly named “ghost tangles” by Braak et al. (Braak, Braak, and Mandelkow [76]) and end-stage dense core plaques. This was also shown in humans where soluble species of Aβ and tau appeared early and correlated well with the clinical diagnosis [34]. Thus, it could be speculated that there are two stages of pathology, one which is dependent on soluble neuropathological lesions where the removal of these might arrest the disease progression, the other is Aβ and tau independent, such that removal of these proteins fails to recover the functional deficits.

### 4.2 Spatial and temporal profiles of neuroinflammation in our mouse models reflects human AD

The observed temporal decrease in the level of gliosis in this study, where the 11–16-month aged cohort of both mouse models showed a higher density of reactive astrocytes and activated microglia compared to the 18–22-month aged cohort, represents the differential immunogenic properties of Aβ and tau species. The 11–16-month-old cohort was correlated with more soluble Aβ and hyperphosphorylated tau aggregates. These soluble aggregates were associated with an elevated neurotoxic inflammatory response which gradually lessened as these soluble aggregates evolved into insoluble dense-core plaques and ghost tangles. This is corroborated from multiple studies in humans, where astrocytosis is prominent in the early phases starting from the preclinical stage and gradually declining throughout disease progression as seen through PET imaging (Carter et al. [78]; Gulyás et al. [79]; Rodriguez-Vieitez et al. [82]). It is important to note, however, that these cellular mediators of neuroinflammation do not undergo proliferation in AD, rather they undergo phenotypic and molecular changes responsible for their prominent presence (Serrano-Pozo et al. [83]). Furthermore, in a few cases of the old age wild-type animals, increased levels of GFAP and clumps of CD68-positive microglia were observed, suggesting that these inflammatory mechanisms might be age-dependent as well. However, the lack of cognitive and behavioural deficits in these mice suggests that there might be two different classes of reactive glial cells, one which is neurotoxic and one which is neuroprotective. A similar classification was proposed by a study adding that these neurotoxic reactive astrocytes are sustained by cytokines released from activated microglia and are found predominantly in the brains of AD patients (Liddelow et al. [81]). Although inflammatory mechanisms are classed as secondary mechanisms, they are crucial in aetiology, as they bridge the gap between cellular alterations in the brain and cognitive and behaviour abnormalities.

Additionaly, levels of Aβ and hyperphosphorylated tau were similar to control in the PRS in both *APP*^*NL-F/NL-F*^ and *APP*^*NL-F*^*/MAPT*^*HTAU*^ mice and different aged cohorts. This was followed by an absence of GFAP^+^ reactive astrocytes and CD68^+^ activated microglia. Similar results were observed in the post-mortem brain tissue of aged AD patients. Thus, our key findings support the hypothesis that the PRS is anatomically preserved in AD, as it does not express cellular and morphological alterations in the fAD mouse models as well as in AD patients. Additionally, the principal excitatory cells of the PRS exhibited preserved functionality in both the *APP*^*NL-F*^*/MAPT*^*HTAU*^ and *APP*^*NL-F/NL-F*^ mice, compared to the principal cells in the CA1 and LEC.

### 4.3 The Intrinsic membrane properties of PRS principal cells are “preserved” but dysfunctional in neighbouring LEC and CA1 regions in both AD mouse models

A prominent feature of the mammalian brain is the heterogeneity of pyramidal cells, and together with the differences in their intrinsic electrical membrane properties, along with the type of synaptic connections they play a crucial role in shaping the dynamics of neuronal networks [11], [12], [9].

We found no significant changes in both AD mouse model genotypes, in the biophysical properties recorded in PRS pyramidal cells; however, we observed significant changes in LEC and CA1 pyramidal cell subthreshold intrinsic membrane properties including, a more depolarized membrane potential, and an increase in membrane input resistance and time constant. This was in contrast to the lack of change of resting membrane potential in CA1 pyramidal cells observed in other Aβ overproducing mouse lines (PSAPP, Tg2576, TAS-TPM) [5], [72]. Studies using the rTg4510 model, which develops hyperphosphorylated tau, neurofibrillary tangles and neurodegeneration ([54]; Spires et al. [85]), revealed altered morphological with depolarization of the resting potential, and increased I_h_-mediated “sag” potentials on injecting negative current pulses in cortical neurons. This was further extended in CA1 of the rTg4510 model, which showed that most of the intrinsic biophysical parameters were unaffected apart from significantly faster membrane time constants, a significant increase in I_h_-mediated sag potential, dendritic and synaptic atrophy was disrupted and associated with altered membrane capacitance correlated with altered behaviour**-**dependent network function [4], [68].

Furthermore, we observed changes in the suprathreshold intrinsic properties, including a decrease in AP threshold and narrower AP waveform in the AD models, although not significantly different than the wild-type AP waveform, others have shown a significantly decreased AP zenith in 9–10 month old PSAPP mice [5], [68] and from *in silico* modelling of a CA1/3 pyramidal neurons, was shown to be depended on increased K^+^ and reduced Na^+^ voltage-gated channels maximal conductance [68].

These alterations in the sub- and suprathreshold membrane properties, culminated in hyperactive membrane properties and an increase in the firing in our AD models observed in LEC and CA1 pyramidal cells; the increase in the firing frequency corroborates previous studies using other AD models [32], [47], [55]. These could be associated with alterations and down regulation of leak conductance responsible for governing the intrinsic firing of these cells. Furthermore, the altered membrane hyperactivity has been suggested to be a result of the changed microenvironment due to pro-inflammatory mediators, such as cytokines, reactive oxygen species and free radicals released from the activated astrocytes and glial cells [47], [55]; this would lead to an altered K^+^ ion homeostasis that would ultimately disrupt excitable membranes, rendering them more hyperactive due an accumulation of extracellular K^+^ released from neurons, a direct impact from the reduced astrocytes function [47], [55], [69]. These alterations in the sub- and suprathreshold membrane properties, culminated in hyperactive membrane properties and an increase in the firing in our AD models observed in LEC and CA1 pyramidal cells; the increase in the firing frequency corroborates previous studies using other AD models [32]. These could be associated with alterations and down regulation of leak conductances responsible for governing the intrinsic firing of these cells. Furthermore, the altered membrane hyperactivity has been suggested to be a result of the changed microenvironment due to pro-inflammatory mediators, such as cytokines, reactive oxygen species and free radicals released from the activated astrocytes and glial cells [47], [55]; this would lead to an altered K^+^ ion homeostasis that would ultimately disrupt excitable membranes, rendering them more hyperactive due an accumulation of extracellular K^+^ released from neurons, a direct impact from the reduced astrocytes function [47], [55], [69].

Overall, the intrinsic membrane properties of PRS principal cells were not altered with age or genotype and correlated with the “preserved” anatomical profile of this region [46], [70]. This is an interesting finding and consistent with a recent study showing that Layer II of EC fan cells, a morphologically distinct principal neuron population, are also resilient to electrophysiological changes, although the majority of the principal neurons, including stellate cells were more excitable at 4 months, despite the expression of intracellular Aβ (iAβ) in the McGill-R-Thy1-APP transgenic rat model [27]. Heggland et al., also showed a similar pattern in dentate gyrus and suggested that Aβ pathology affects the entorhinal-hippocampal network transiently and selectively, a region that is more vulnerable during AD pathogenesis.

The resilience of these cells will have a significant impact on circuit behaviour, since the firing pattern of a neuron tells us the type of ion channels it possesses and the way it will behave during network activity; for example, the high-frequency burst firing pyramidal cells of the PRS arriving at presynaptic terminals, will increase the probability of synaptic release per burst and would favour a high gain, synaptic facilitation [29], [37], whereas tonic, regular firing cells would function to attenuate prolonged excitatory stimuli, while transferring phasic responses more favourably and would be efficient in synapses that display synaptic depression. These concepts of the functional consequences of presynaptic firing rates have been modelled previously [40]. Bursting activity of neurons is thought to be associated with consummatory behaviour and network-driven during sleep [65], [66], [8], [9], and more recently, the significance of bursting in the subiculum, a relay centre for the processed information of the hippocampus, is thought to carry more spatial information than tonic firing cells [62]. Furthermore, increases in persistent firing are thought to be implicated in successful learning ability, whereas a decrease is thought to contribute to learning impairments in aging [36].

Given the parallels of the subiculum with the PRS in its role as a major output of the hippocampus, in combination with its dual entorhinal and CA1 input, implies that the PRS may play a role in gating information flow out of the hippocampus, and thus the strong burst firing activity of the PRS cells will result in high efficacy information transfer, ensuring that the processed information is reliable and unaffected by Aβ and tau pathology.

In contrast, the altered firing properties of pyramidal cells observed in the LEC and CA1 will have detrimental effects in the intrinsic signalling within the neuron, as high-frequency burst backpropagation into the dendrites has been shown to influence synaptic integration and pre-/postsynaptic coincidence detection in ways distinct from a single action potential.

Moreover, the altered firing will have profound implications for the efficacy of information transfer between neurons. For example, previously, we showed that early stage disrupted intrinsic firing of pyramidal cells of the LEC was correlated with the dysfunction of molecular and synaptic outputs in *APP*^*NL-F/NL-F*^ mice, preceding the typical hallmarks of AD [50], which is also corroborated by others using the rTg4510 transgenic mouse line studying tauopthay [30] and in a double transgenic amyloid (App23, PS45) AD mouse model [6], as well as studies that showed exposure to high doses of Aβ toxic species (800 nM–10 μM), that resulted in neuronal hyperexcitability triggering progressive epilepsy [41]. We suggested that initial aberrant synaptic hyperactivity originated from a decreased cellular distribution of GABAergic function, which in later studies, was shown to be interneuron-subtype selective destruction [41].

It would be beneficial to further understand the mechanisms that alter the intrinsic membrane properties in LEC and CA1 and not in PRS pyramidal cells, which could provide us with a basis to modify disease progression in brain regions that are severely affected by the disease. For example, dysfunctional synaptic activity has been shown to promote the spread of tau; in vitro and in vivo models of AD have demonstrated that increased neuronal activity stimulates tau release which further enhances tau pathology (Wu et al. [87], [88]) as well as spread of Aβ [73], consistent with preclinical human fMRI studies that have also shown pathology to be initiated in the LEC [33].

### 4.4 Conclusions

In summary, we report here that the PRS is anatomically and functionally preserved in two AD mouse models, with a lack of neuropathological lesions, inflammatory markers and neuronal loss. This could have implications in preventing the spread of tau seeds and progression of Aβ plaques such that the continuum of AD could be disrupted at an early stage before the behavioural and cognitive deficits are precipitated. Future work is needed to further identify cellular pathways underlying the PRS resilience, revealing important biological markers and is ongoing by combining various expertise, including histopathology, synaptic physiology, and molecular profiling of tissue from human AD patients and these physiologically relevant AD mouse models used in this study that recapitulate human AD pathology.

## Competing interests statement

There are no competing interests.

## CRediT authorship contribution statement

Anam Islam: Performed all neuroanatomical studies and analysed the data and contributed in preparing the manuscript. Takashi Saito: Designed and provided the mouse models. Takaomi C Saido: Designed and provided the mouse models. Afia B. Ali: Designed and coordinated the project, performed all electrophysiological whole-cell recordings, reconstructed cells, supervised neuroanatomical studies, performed data analysis, and prepared the manuscript.
